# Bulk and single-cell transcriptome analysis reveal shared key genes and patterns of immune dysregulation in systemic lupus erythematosus and sepsis

**DOI:** 10.1186/s10020-025-01350-y

**Published:** 2025-12-30

**Authors:** Xuehuan Wen, Songjie Bai, Kangwei Sun, Kai Zhang, Ruomeng Hu, Shumin Li, Jie Yang, Lanxin Cao, Zhijian Cai, Gensheng Zhang

**Affiliations:** 1 Department of Oncology, The People’s Hospital of Cangnan Zhejiang, Wenzhou Medical University, Wenzhou, Zhejiang 325800 China; 2 Department of Critical Care Medicine, The Second Affiliated Hospital of Zhejiang University School of Medicine, Hangzhou, Zhejiang 310009 China; 3 Department of Emergency Medicine, Dongyang People’ Hospital of Wenzhou Medical University, Zhejiang, 322100 China; 4 Department of Respiratory and Critical Care Medicine, The First Affiliated Hospital of Wenzhou Medical University, Wenzhou, Zhejiang 325000 China; 5 Department of Orthopedics of the Second Affiliated Hospital, Institute of Immunology, Zhejiang University School of Medicine, Hangzhou, 310009 China; 6 Ministry of Education, Key Laboratory of Multiple Organ Failure (Zhejiang University), Hangzhou, Zhejiang 310009 China

**Keywords:** Sepsis, Systemic lupus erythematosus, *PLSCR1*, Single-cell transcriptome, CD14^+^ monocyte

## Abstract

Systemic lupus erythematosus (SLE) patients often experience sepsis, a significant clinical challenge that frequently requires intensive care and contributes significantly to morbidity and mortality. However, the molecular mechanisms underlying this comorbidity remain poorly understood. This study investigated the molecular intersection of SLE and sepsis using publicly available gene expression datasets. We identified 93 differentially expressed genes (DEGs) (49 upregulated and 44 downregulated) shared by both conditions, primarily impacting bacterial defense and immune response pathways. Integrated differential expression and weighted gene co-expression network analyses (WGCNA) revealed 11 hub genes, with TNFAIP6 and PLSCR1 showing significant diagnostic potential. Single-cell RNA sequencing (scRNA-seq) of peripheral blood mononuclear cells (PBMCs) revealed a significant expansion of CD14^+^ monocytes in both conditions, exhibiting elevated expression of the identified hub genes and similar transcriptional profiles. Upregulation of *TNFAIP6* and *PLSCR1* was validated using public monocyte datasets and real-time PCR of mouse PBMCs, with PLSCR1 showing markedly increased expression in SLE cases complicated by sepsis. Our findings demonstrate that CD14^+^ monocytes emerge as a common site of immune dysregulation in these comorbid conditions, with overlapping gene expression and pathway alterations suggesting shared molecular mechanisms that could inform targeted therapeutic strategies.

**Supplementary Information:**

The online version contains supplementary material available at 10.1186/s10020-025-01350-y.

## Introduction

Systemic lupus erythematosus (SLE) is a complex chronic autoimmune disease characterized by the production of autoantibodies and systemic inflammation, leading to potential damage across multiple organ systems, including the kidneys, heart, and brain [1]. This multi-organ involvement contributes significantly to morbidity and mortality through complications such as lupus nephritis, pericarditis, and neuropsychiatric syndromes [1, 2]. Predominantly affecting young women, SLE has a global incidence ranging from 1.5 to 11 cases per 100,000 people [3]. The etiology of SLE is multifactorial, involving an interplay between genetic susceptibility, environmental factors, and diverse immunological dysfunctions [4–6]. Standard therapeutic approaches rely on glucocorticoids, antimalarials, and broad immunosuppressants to manage disease activity [7]. While these treatments have improved survival rates over recent decades [8, 9], the requisite long-term immunosuppression significantly increases patient vulnerability to infections [10]. Indeed, infections represent a leading cause of death in SLE populations [11], with sepsis being the primary driver for intensive care unit (ICU) admissions and associated mortality in these patients [12].

Sepsis, defined as a life-threatening organ dysfunction caused by a dysregulated host response to infection, frequently necessitates critical care interventions [13, 14]. Individuals with SLE exhibit heightened susceptibility to sepsis, attributed to both intrinsic immune abnormalities associated with the disease and treatment-induced immunosuppression, often compounded by hematological alterations like leukopenia and thrombocytopenia [10, 15, 16]. Despite the clear clinical link and the shared feature of immune dysregulation in both SLE and sepsis, the common pathophysiological mechanisms connecting these conditions remain poorly understood. Elucidating shared molecular and cellular alterations could provide crucial insights for developing targeted diagnostic tools and more effective therapeutic strategies, ultimately improving outcomes for this vulnerable patient group.

To bridge this knowledge gap, our study employed an integrative approach, merging bioinformatics with experimental validation. By analyzing patient samples, we identified crucial shared molecular signatures linking SLE and sepsis. This led us to pinpoint promising candidate biomarkers and highlighted CD14^+^ monocytes as key cellular players in this disease crosstalk. Subsequent validation studies, focusing on comparative expression analysis in relevant ex vivo and in vivo models, corroborated these bioinformatic predictions. These observations provide further evidence supporting potential molecular links that may contribute to the heightened sepsis susceptibility observed in SLE patients.

## Methods

### Data collection and processing

**Fig. 1:**
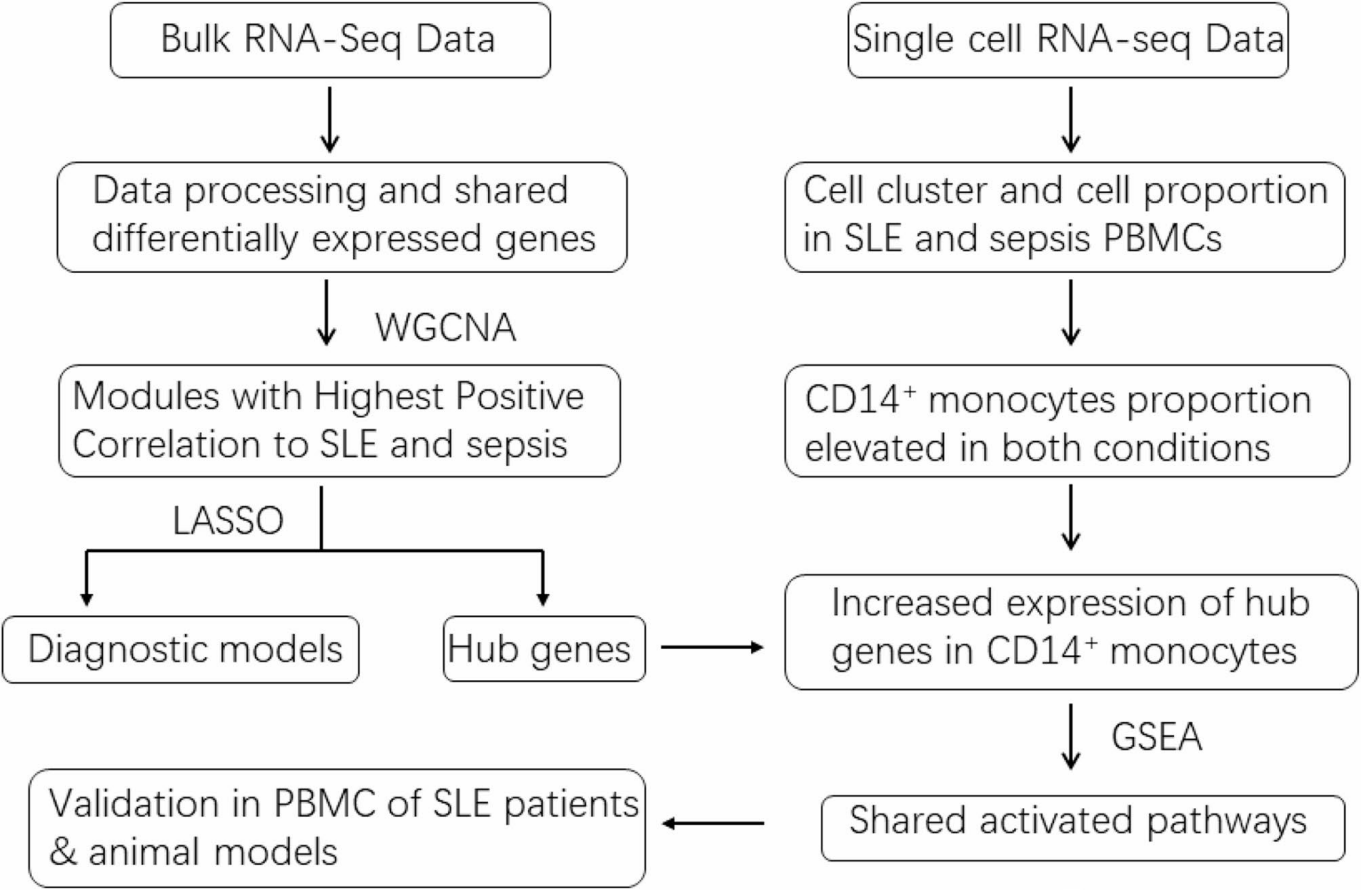
The flow chart of this study. GEO, Gene Expression Omnibus; SCP, Single Cell Portal; DEGs, differential expressed genes; SLE, Systemic lupus erythematosus; WGCNA, weighted gene co-expression network analysis; LASSO, Least absolute shrinkage and selection operator

Our integrative analysis utilized multiple publicly available transcriptomic datasets from the NCBI Gene Expression Omnibus (GEO) database and The Broad Institute Single Cell Portal. For initial signature identification, we analyzed two sepsis datasets (GSE95233 [17], GSE57065 [18]) and two SLE datasets (GSE61635 [19], GSE49454 [20]) to identify differentially expressed genes and potential shared molecular signatures. To explore cellular heterogeneity, we examined scRNA-seq data from GSE135779 [21] (peripheral blood mononuclear cells (PBMCs) from 40 SLE patients and 16 healthy controls) and SCP548 (PBMCs from 29 sepsis patients and 19 healthy controls). For validation, we further investigated the expression profiles of our prioritized candidate genes, *TNFAIP6* and *PLSCR1*, in independent human monocyte datasets GSE46955 [22] (sepsis vs. healthy) and GSE148601 (SLE vs. healthy), as well as assessed their expression changes in PBMCs from SLE patients before and after ex vivo LPS stimulation using GSE231686 [23]. To correlate with disease severity, we incorporated GSE185263 [24] for sepsis severity analysis and GSE228066 [25] for SLE disease activity analysis. This comprehensive analytical workflow is illustrated in Fig. 1, with detailed dataset information summarized in Table 1 and patients’ demographic details provided in supplementary Tables 1 and 2.

**Table 1:** The source information of sepsis and SLE datasets

Accession ID	Platform	Tissue	Total	Patient	Healthy Control
Sepsis					
GSE95233	GPL570	Peripheral Blood	124	102	22
GSE57065	GPL570	Peripheral Blood	107	82	25
SCP548	Unknown	PBMC	48	29	19
GSE46955	GPL6104	Monocyte	14	8	6
GSE185263	GPL16791	Peripheral Blood	392	348	44
SLE					
GSE61635	GPL570	Peripheral Blood	129	99	30
GSE49454	GPL10558	Peripheral Blood	177	157	20
GSE135779	GPL20301	PBMC	56	40	16
GSE148601	GPL17077	Monocyte	26	12	14
GSE231686	GPL20795	PBMC	32	32	0
GSE228066	GPL28038	PBMC	45	45	0

### Identification of DEGs

We obtained the normalized gene count tables from the GSE95233, GSE57065, GSE61635, and GSE49454 datasets. The probe IDs of the microarray were converted into gene symbols using the “tinyarray” package in R. With the aid of the ‘limma’ package in R, differential expression analysis was conducted on GSE95233 for sepsis versus healthy controls and GSE61635 for SLE versus healthy controls [26]. Genes with |log2 Fold change| >1 and adjusted *P*-value < 0.05 were considered as differentially expressed.

### Gene ontology (GO) analysis

The common DEGs were converted to gene IDs using the R package “org.Hs.eg.db”. Analyses of GO for DEGs were performed by the R package “clusterProfiler” [27]. The significantly different GO terms and signal pathways were screened by the threshold *P*-value < 0.05. The top five enriched terms from the Biological Process (BP) and Cellular Component (CC) categories, as well as the sole enriched term from the Molecular Function (MF) category, were visualized using the ‘enrichplot’ and ‘ggplot2’ packages in R.

### WGCNA

WGCNA was performed on the GSE95233 and GSE61635 datasets using the “WGCNA” package in R software to identify correlated gene modules in sepsis and SLE. During the WGCNA analysis of GSE61635, three outlier specimens (GSM1509664, GSM1509669, and GSM1509698) were identified and excluded due to their extreme expression profiles. A co-expression network was then constructed by setting a soft threshold power to ensure a high scale-free topology fitting index (R^2^) and mean connectivity among the genes. The minimum number of gene modules in the dynamic tree cut and module identification section was set to 100. Clinical characteristic data for sepsis and SLE samples were obtained from the GSE95233 and GSE61635 datasets, and the relationship between gene modules and clinical traits was visualized in a heatmap.

### LASSO regression

To evaluate the efficacy of the 11 pre-selected genes in diagnosing SLE, we employed LASSO regression using the “glmnet” package in R [28]. We fit the LASSO model to the training dataset GSE61635 and performed 5-fold cross-validation using the glmnet function to determine the optimal tuning parameter (λ) that minimizes the mean cross-validated error. The final LASSO model included two of the 11 genes with non-zero coefficients, which were considered essential predictors of SLE disease status based on the model.

### ROC curve

To assess the performance of the LASSO model in diagnosing SLE, ROC curve analyses were conducted on gene expression data from GSE61635 and GSE49454 datasets using the pROC package [29]. Simultaneously, ROC curve analyses were performed on the GSE95233 and GSE57065 datasets to evaluate the diagnostic value of crucial genes in sepsis. The area under the curve (AUC) was utilized to compare the discriminative abilities of the LASSO models in diagnosing SLE and the individual genes in diagnosing sepsis.

### Analysis of Single-Cell RNA sequencing data

Criteria applied to the GSE135779 dataset included the number of feature RNA between 200 and 2500, UMI count ≥ 400, and mitochondrial gene percentage < 0.2. The following criteria were applied to the SCP548 dataset: number of feature RNA between 500 and 3000, UMI count > 500, and mitochondrial gene percentage < 0.15. The Seurat software package (version 4.3.0, https://satijalab.org/seurat/) was employed to normalize the expression matrix and obtain scaled data. The principal component analysis (PCA) was constructed using the top 2,000 highly variable genes for sample integration via the harmony package [30]. Construction of Uniform Manifold Approximation and Projection (UMAP) was based on the top 30 harmony components. The UCell package was utilized to calculate the enrichment score of the hub genes across all cells [31]. We performed a differential expression analysis with the FindMarkers function in Seurat to identify the DEGs in different disease states. We screened for DEGs using the following thresholds: |average fold change| ≥ 0.25, *P*-value ≤ 0.05.

### Functional enrichment analysis of key genes

To elucidate the functions of DEGs identified in monocytes from sepsis or SLE patients compared to healthy controls, we performed Gene Set Enrichment Analysis (GSEA). Hallmark pathways with significant enrichment were ranked and visualized based on the Net Enrichment Score (NES), gene counts, and *P*-value.

### Ethical approval

The animal research was approved under Ethics Lot Number ZJU20230388 by the Ethics Committee of the Laboratory Animal Center of Zhejiang University, Hangzhou, China.

### Animal models and experimental procedures

Female BALB/c mice (8–10 weeks old) from Shanghai SLAC Laboratory Animal Co., Ltd. (Shanghai, China) were housed under specific pathogen-free conditions at Zhejiang University’s Laboratory Animal Center (Hangzhou, China). Mice were maintained at 22 ± 2 °C, 55 ± 10% humidity, with a 12-hour light/dark cycle and ad libitum access to standard chow and sterile water. The study comprised four distinct experimental groups: Control (healthy mice receiving PBS), SLE (mice with pristane-induced lupus), Sepsis (healthy mice with LPS-induced sepsis), and SLE + Sepsis (lupus mice with LPS-induced sepsis). For the SLE and SLE + Sepsis groups, mice received a single intraperitoneal (i.p.) injection of 0.5 ml pristane (Meilunbio, Dalian, China) to induce SLE-like autoimmunity [32]. Six months post-pristane administration, acute sepsis was induced in the Sepsis and SLE + Sepsis groups by i.p. injection of lipopolysaccharide (Solarbio Science & Technology Co., Ltd., Beijing, China) at 20 mg/kg body weight, while Control and SLE groups received an equivalent volume of sterile PBS. LPS dose was selected to elicit a robust acute systemic inflammatory response [33]. Twelve hours post-injection, mice were euthanized and peripheral blood was immediately collected via cardiac puncture into EDTA-coated tubes. Lung and liver tissues were harvested for hematoxylin and eosin (H&E) staining to confirm successful model establishment. PBMCs were isolated using a Mouse Peripheral Blood Lymphocyte Separation Kit (Beyotime Biotechnology, Shanghai, China) according to the manufacturer’s protocol for subsequent analyses.

### Real-time PCR

Using TRIzol reagent (Thermo Fisher Scientific), we isolated total RNA according to the provided guidelines. cDNA was generated using a synthesis kit (cwbio, China), in accordance with the manufacturer’s suggested protocols. For the purpose of mRNA quantification, β-actin was employed as an endogenous reference. Real-time PCR assays were performed on an Applied Biosystems 7500 system (Thermo Fisher Scientific) using SYBR Green (tsingke). The specific primers used for these assays are detailed in Table 2.

**Table 2:** Primer sequences

Name	Primer sequence (5′→3′)
β-Actin	F, GGCTGTATTCCCCTCCATCG; R, CCAGTTGGTAACAATGCCATGT
Tnfaip6	F, GGGATTCAAGAACGGGATCTTT; R, TCAAATTCACATACGGCCTTGG
Plscr1	F, GGTATCCCCCTCCGTATCCAC; R, GCCACCACCTGCATAACCT

*F* Forward, *R* Reverse

### Statistical analysis

The statistical component of our research was conducted using the R software (version 4.2.2). Differences in gene expression among groups were statistically tested using the Wilcoxon test or t-test, with a *P*-value of less than 0.05, denoting statistical significance. The correlations within our data were evaluated using Pearson or Spearman correlation analysis.

## Results

### Identification of common DEGs and GO analysis in sepsis and SLE

**Fig. 2:**
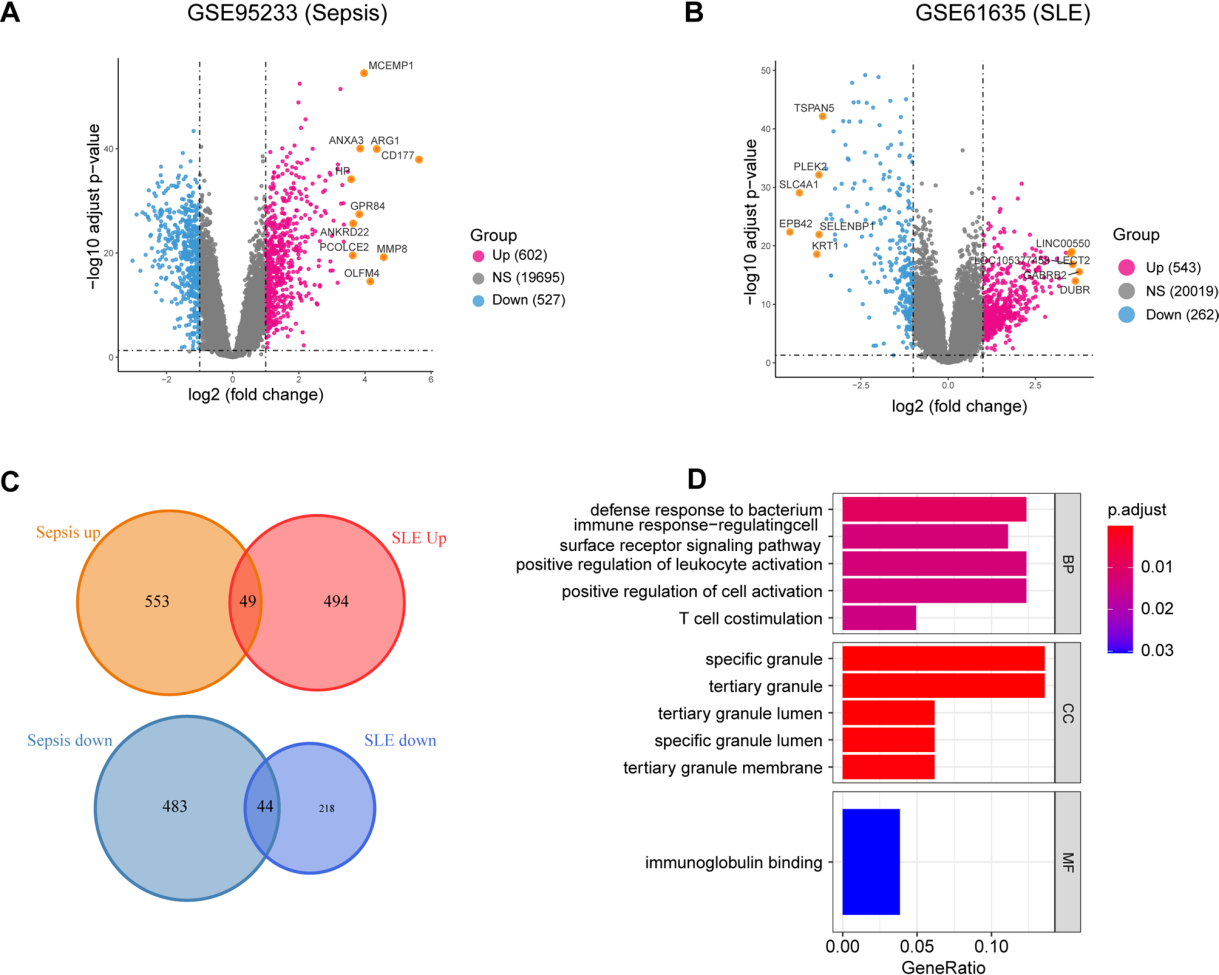
Identification of Common DEGs between Sepsis and SLE. (**A**) Volcano plot of DEGs within sepsis dataset GSE95233. (**B**) Volcano plot of DEGs within SLE dataset GSE61635. (**C**) The intersections in dual Venn diagrams represent shared upregulated (top) and downregulated (bottom) genes between SLE and sepsis datasets. (**D**) GO enrichment outcomes for the common DEGs. DEGs, differentially expressed genes; BP, biological process; CC, cellular component; MF, molecular function

We analyzed datasets GSE95233 and GSE61635 to identify DEGs using an absolute log2 fold change (log2FC) cut-off of 1 and an adjusted *P*-value threshold of 0.05. In the sepsis cohort, we identified 1129 DEGs—602 upregulated and 527 downregulated (Fig. 2A). For SLE, we detected 805 DEGs, consisting of 543 upregulated and 262 downregulated genes (Fig. 2B). A comparative analysis revealed 49 genes that were co-upregulated and 44 that were co-downregulated in both conditions (Fig. 2C). To ascertain the functional implications of these DEGs, we conducted GO enrichment analysis. The five most significantly enriched GO categories are presented in a bar chart and ranked by *P*-value. In the category of biological process (BP), the majority of the enrichment was related to defence against infection and immune regulation. The cellular component (CC) category primarily involved granular structures, while the only significantly enriched term in the molecular function (MF) category was immunoglobulin binding (Fig. 2D).

### Identification of modules with highest positive correlation to disease states

**Fig. 3:**
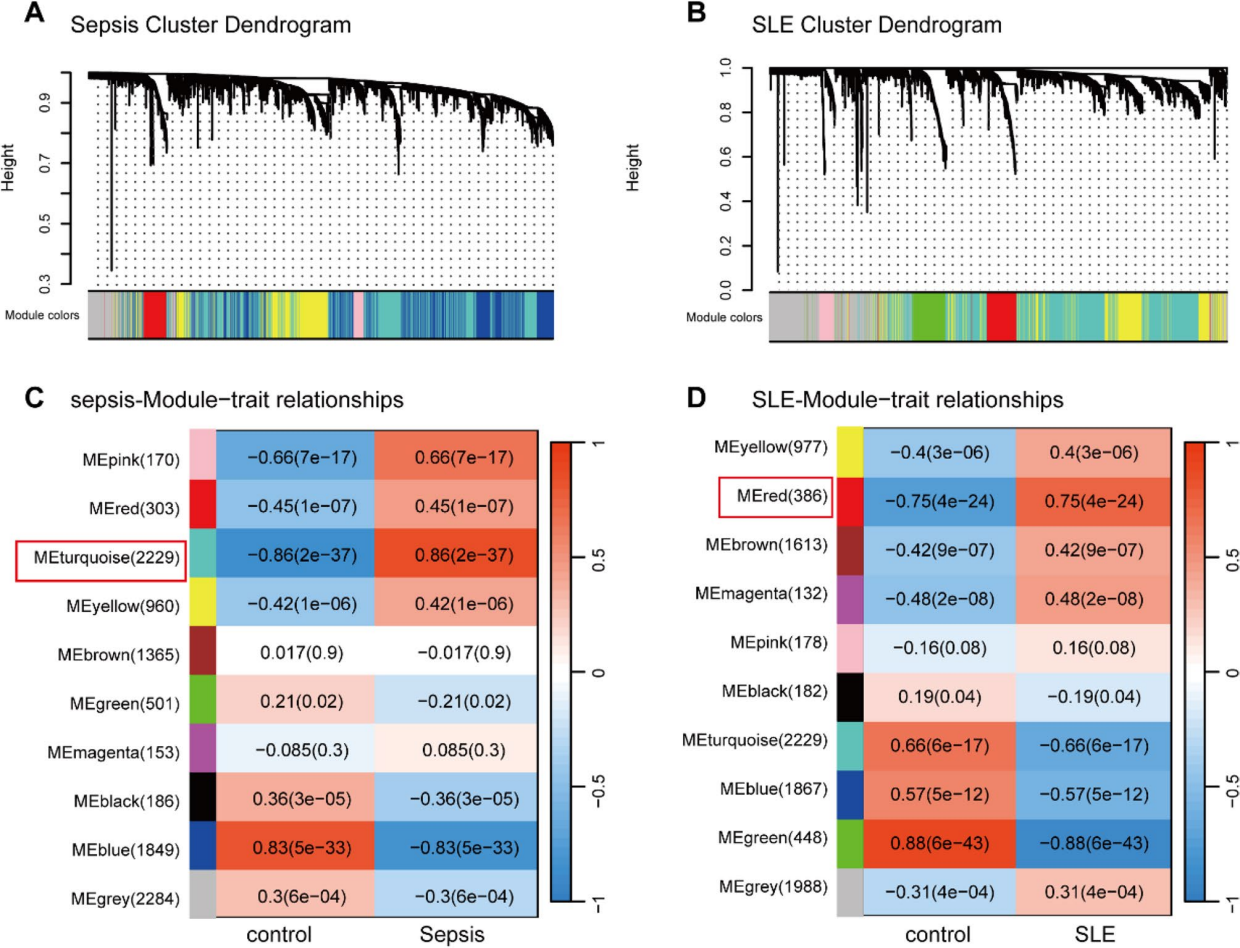
Identification of modules correlated to clinical traits of sepsis and SLE by WGCNA.** (A**,** B)** Cluster dendrogram of co-expressed genes in sepsis **(A)** and SLE **(B)**, various colours represent different modules. **(C**,** D)** Heatmap of module–trait relationships in sepsis **(C)** and SLE **(D)**. Each row corresponds to a module, and each column corresponds to a clinical trait. Each cell contains a corresponding correlation and *P*-value of modules with various clinical traits. SLE, Systemic lupus erythematosus; WGCNA, weighted gene co-expression network analysis

We constructed a scale-free topology network using a soft threshold 8 for GSE95233 (Supplementary Fig. 1A). Similarly, we set a soft threshold of 10 for GSE61635 to build a scale-free network (Supplementary Fig. 1B). Following this, a gene cluster tree was generated for both datasets, using hierarchical clustering analysis based on adjacency value differences. This identified modules containing a minimum of 100 genes (Fig. 3A, B). To elucidate the relationships between these modules and the disease states, heatmaps were generated using Pearson correlation coefficients. In the WGCNA analysis of the GSE95233 dataset, the turquoise module displayed the strongest positive correlation with sepsis (*r* = 0.86, *P *= 2e-37, genes = 2229) (Fig. 3C). Similarly, for the GSE61635 dataset, the red module exhibited the highest positive association with SLE (*r* = 0.75, *P*= 4e-24, genes = 386) (Fig. 3D).

### Significant diagnostic value of TNFAIP6 and PLSCR1 in sepsis and SLE

**Fig. 4:**
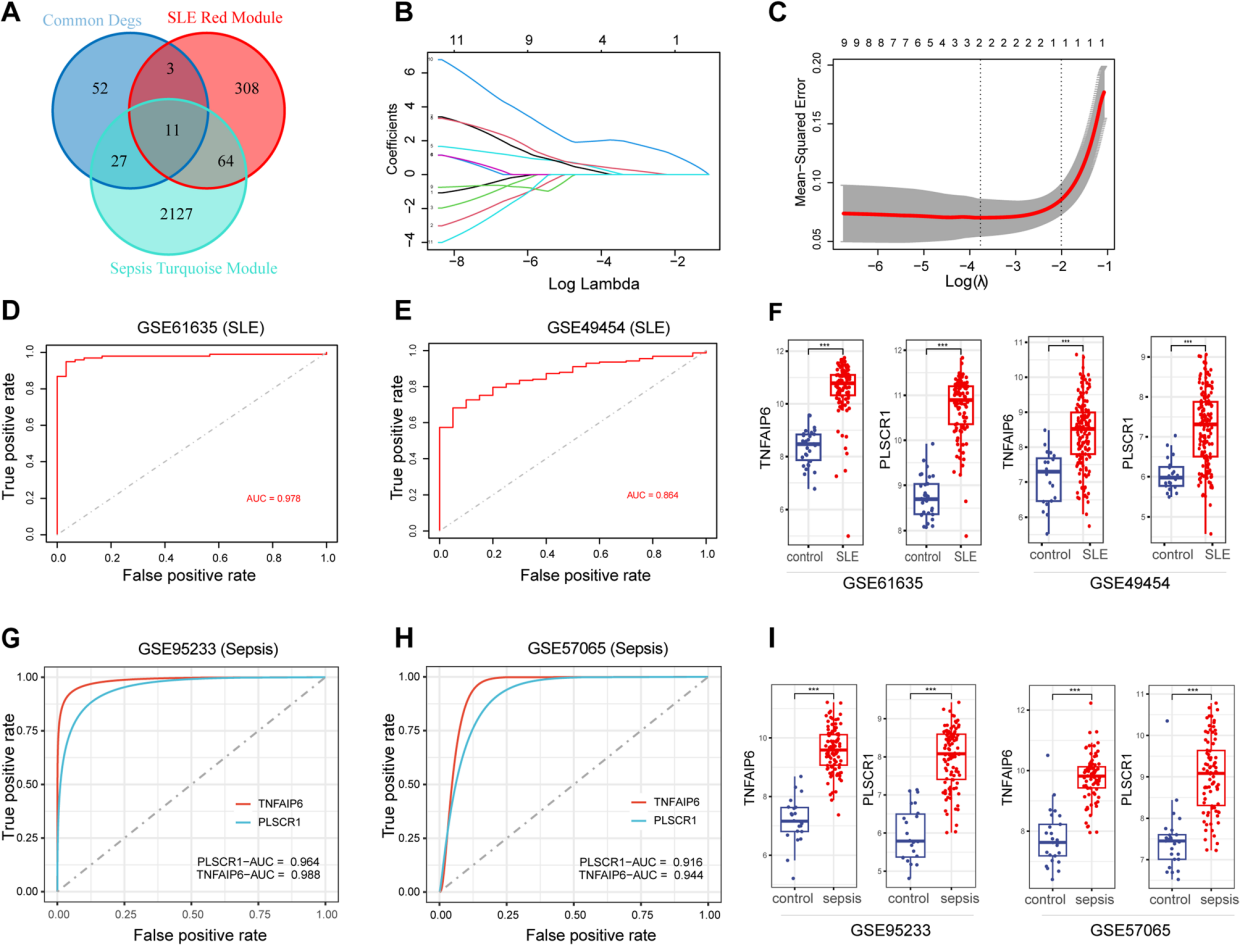
LASSO Construction of diagnostic signatures with hub genes for SLE and sepsis. (**A**) Venn diagram representing the central genes that are co-expressed in DEGs and modules with the highest positive correlation in SLE and sepsis datasets. (**B**) LASSO regularization paths for the predictive genes. The plot shows the LASSO coefficients of each gene against the log-scaled λ value. Each line represents one gene. (**C**) Cross-validated Mean Square Error (MSE) for the Lasso model. The plot shows the average MSE against the log-scaled λ value. The dotted vertical line at “min” denotes the λ value with the minimum MSE. (**D**) ROC curve shows AUC values of the LASSO model for predicting SLE onset in training data GSE61635. (**E**) ROC curve shows AUC values of the LASSO model for predicting SLE onset in test data GSE49454. (**F**) The expression profiles of *TNFAIP6* and* PLSCR1* in dataset GSE61635 and external SLE dataset GSE49454. (**G**) ROC curve shows AUC values of the *TNFAIP6* and* PLSCR1* for predicting sepsis onset in GSE95233. (**H**) ROC curve shows AUC values of the *TNFAIP6* and *PLSCR1* for predicting sepsis onset in GSE57065. (**I**) The expression profiles of *TNFAIP6* and *PLSCR1* in dataset GSE95233 and external sepsis dataset GSE57065. Statistical significance was determined using unpaired Wilcoxon rank-sum test (***P < 0.001)

To identify key genes implicated in the pathophysiology of both sepsis and SLE, we conducted an intersection analysis between the common DEGs identified in both conditions and previously defined sepsis-related and SLE-related gene modules (Fig. 4A). This strategy yielded 11 candidate genes: *ANKRD22*, *RSPH9*, *DHRS9*, *AIM2*, *CCNA1*, *CEACAM1*, *FBXO6*, *TNFAIP6*, *FCGR1A*, *PLSCR1*, and *FCGR1BP*. These genes were significantly upregulated in sepsis and SLE (Supplementary Fig. 2A, B). Subsequently, these 11 genes underwent LASSO regression analysis using the GSE61635 dataset to construct a SLE diagnostic signature. The optimal regularization parameter (λ) was determined by 5-fold cross-validation. This process identified two genes, *PLSCR1* and *TNFAIP6*, as the essential predictors in the LASSO model, as they elicited non-zero coefficients (Fig. 4B and C). The diagnostic performance of this two-gene signature was evaluated using ROC curve analysis within the training dataset (GSE61635), achieving an AUC of 0.978, indicating excellent discriminatory ability for SLE (Fig. 4D). To assess the model’s predictive ability in an external dataset, we performed ROC curve analysis using the validation SLE dataset GSE49454. The resulting AUC value of 0.864 in this validation cohort further substantiated the diagnostic utility of the PLSCR1 and TNFAIP6 signature for SLE (Fig. 4E). Furthermore, the expression levels of *TNFAIP6* and *PLSCR1* were significantly elevated in SLE patients compared to healthy individuals in both the GSE61635 and GSE49454 datasets (Fig. 4F).

Considering that a diagnostic model derived from SLE data may not be directly transferable to sepsis, we next evaluated the individual diagnostic potential of *TNFAIP6* and *PLSCR1* for sepsis. ROC curve analyses were performed using the GSE95233 sepsis dataset to assess the sensitivity and specificity of each gene independently. Both *TNFAIP6* and *PLSCR1* demonstrated substantial diagnostic relevance for sepsis, each achieving AUC values exceeding 0.90 (Fig. 4G-H). Moreover, paralleling the observations in SLE, the transcript levels of both TNFAIP6 and PLSCR1 were significantly higher in sepsis patients relative to controls within the primary sepsis dataset (GSE95233) and were confirmed in an independent sepsis validation cohort (GSE57065) (Fig. 4I). Collectively, these findings highlight the significant diagnostic value of *TNFAIP6* and *PLSCR1* in both SLE and sepsis.

To investigate the association between gene expression and disease severity, we performed Spearman correlation analyses. In the SLE cohort (GSE228066), *PLSCR1* expression showed a significant positive correlation with the SLE Disease Activity Index (SLEDAI) score (Spearman’s ρ = 0.36, *P* = 0.016, Supplementary Fig. 2 C). Conversely, no significant correlation was observed between *TNFAIP6* expression and SLEDAI score (ρ = −0.005, *P *= 0.98, Supplementary Fig. 2D). In the sepsis cohort (GSE185263), expression levels of both PLSCR1 (ρ = 0.12, *P* = 0.025, Supplementary Fig. 2E) and TNFAIP6 (ρ = 0.11, * P* = 0.041, Supplementary Fig. 2 F) were significantly, albeit modestly, positively correlated with the Sequential Organ Failure Assessment (SOFA) score. These findings suggest that the expression of these genes correlates with disease severity markers, potentially indicating their involvement in the pathophysiology of both conditions.

### CD14^+^ monocyte expansion and associated hub gene dysregulation correlate with disease activity in SLE

**Fig. 5:**
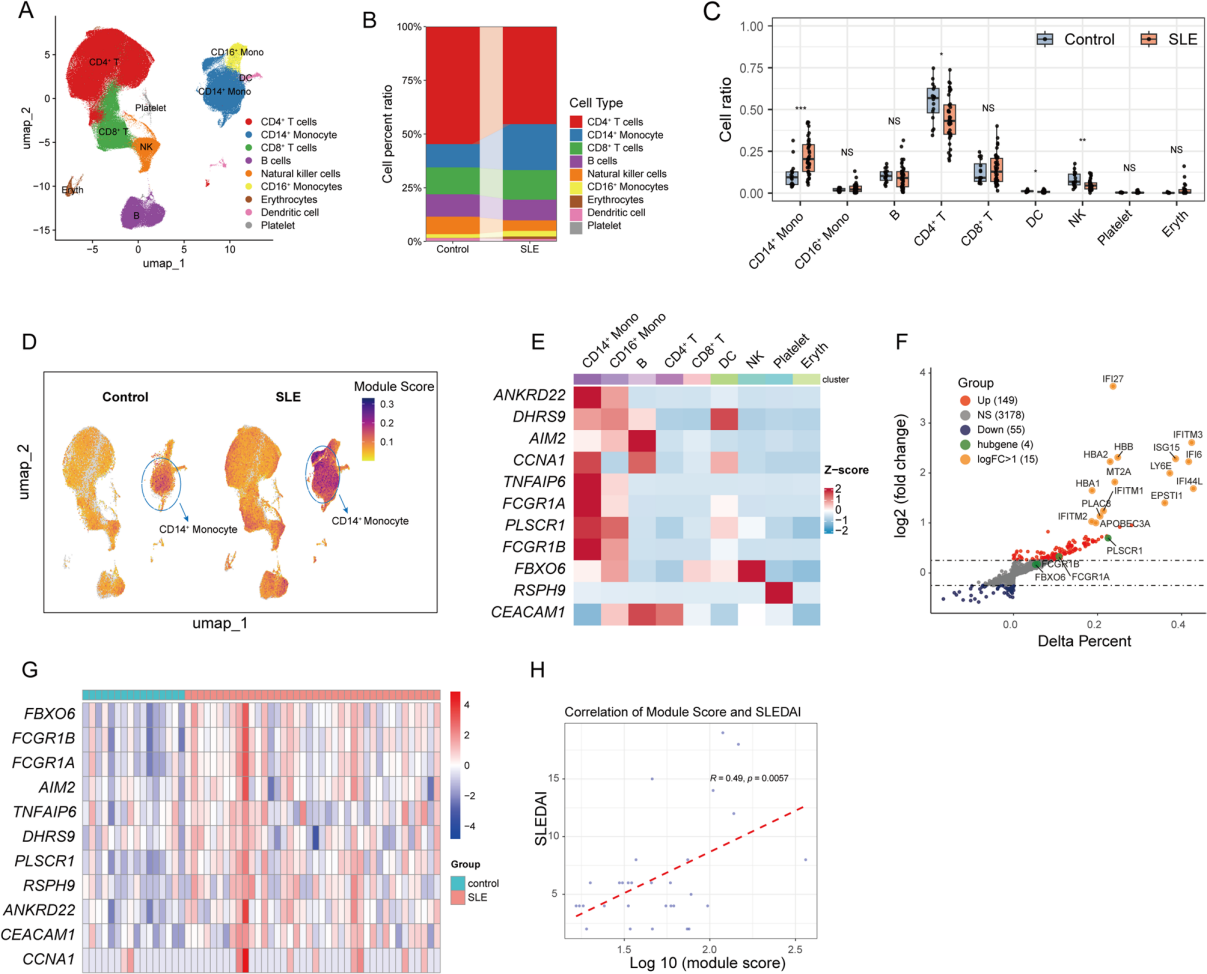
scRNA-seq Reveals Increased CD14^+^ Monocytes Proportion and Hub Gene Overexpression in SLE.** (A)** UMAP plots for each cell type, coloured by different cell types. **(B)** The average cell proportions in PBMCs from SLE patients and healthy controls. **(C)** Comparison of cell type proportions across all samples, grouped by disease status. Each point illustrates the proportion of a particular cell type in a given sample. **(D)** Module score distribution in UMAP space for eleven hub gene modules evaluated using UCell, and grouped by disease states. **(E)** Heatmap showing the average expression of eleven hub genes between all cell types. The colour scale corresponds to z-score. **(F)** Volcano plot displaying the results from differential expression analysis using the Wilcoxon rank-sum test between CD14^+^ monocytes from SLE patients and healthy controls. The X-axis signifies the difference in individual gene detection proportions between the SLE and control groups. Genes with log2(FC) > 1 are highlighted in orange. **(G)** Heatmap showing the expression of eleven hub genes across all samples. The colour scale corresponds to z-score. **(H)** The correlation between SLEDAI and log10(module score). Statistical significance was determined using unpaired Wilcoxon rank-sum test (*P < 0.05, **P < 0.01, ***P < 0.001, NS, not significant)

To elucidate cell type-specific gene expression patterns in peripheral blood, we analyzed publicly available scRNA-seq data (GSE135779) derived from PBMCs of SLE patients and healthy controls. Following standard data processing pipelines, including quality control, normalization, and clustering, the cells were clustered into 21 clusters (Supplementary Fig. 3A). Annotation based on canonical marker gene expression subsequently identified nine major cell populations (Fig. 5A, Supplementary Fig. 3B). Significant alterations in cellular proportions were observed between SLE patients and healthy controls, most notably a marked increase in the relative abundance of CD14^+^ monocytes (*P* < 0.001), alongside differences in CD4^+^ T cells and NK cells (Fig. 5B, C). To investigate the expression patterns of the 11 previously identified hub genes across these cell populations, we employed the UCell package to calculate module scores. Notably, CD14^+^ monocytes from SLE patients displayed the highest aggregate module scores (Fig. 5D), an observation corroborated by heatmap visualization, which highlighted the enriched expression of these hub genes within this specific cell type (Fig. 5E).

Consequently, subsequent analyses focused specifically on the CD14^+^ monocyte population. Differential gene expression analysis comparing CD14^+^ monocytes between SLE patients and controls identified 149 upregulated and 55 downregulated genes (applying criteria of |average log2 Fold Change| >0.25 and adjusted *P*-value < 0.05). Among the most significantly upregulated DEGs, *PLSCR1* and *FCGR1A* exhibited substantial increases in expression levels in SLE monocytes compared to controls (Fig. 5F). Furthermore, examination of the individual expression levels of all 11 hub genes within CD14^+^ monocytes across all samples consistently revealed significantly higher expression in the SLE group compared to the control group (Fig. 5G).

Finally, we assessed the potential clinical relevance of these findings by correlating the cumulative hub gene module scores in CD14^+^ monocytes with the Systemic Lupus Erythematosus Disease Activity Index (SLEDAI). This analysis revealed a moderate but statistically significant positive correlation (Pearson’s *R* = 0.49, *P* = 0.0057), suggesting a potential association between the activity of these hub genes in CD14^+^ monocytes and SLE disease severity (Fig. 5H).

### Altered hub gene signature in expanded CD14^+^ monocytes characterizes sepsis

**Fig. 6:**
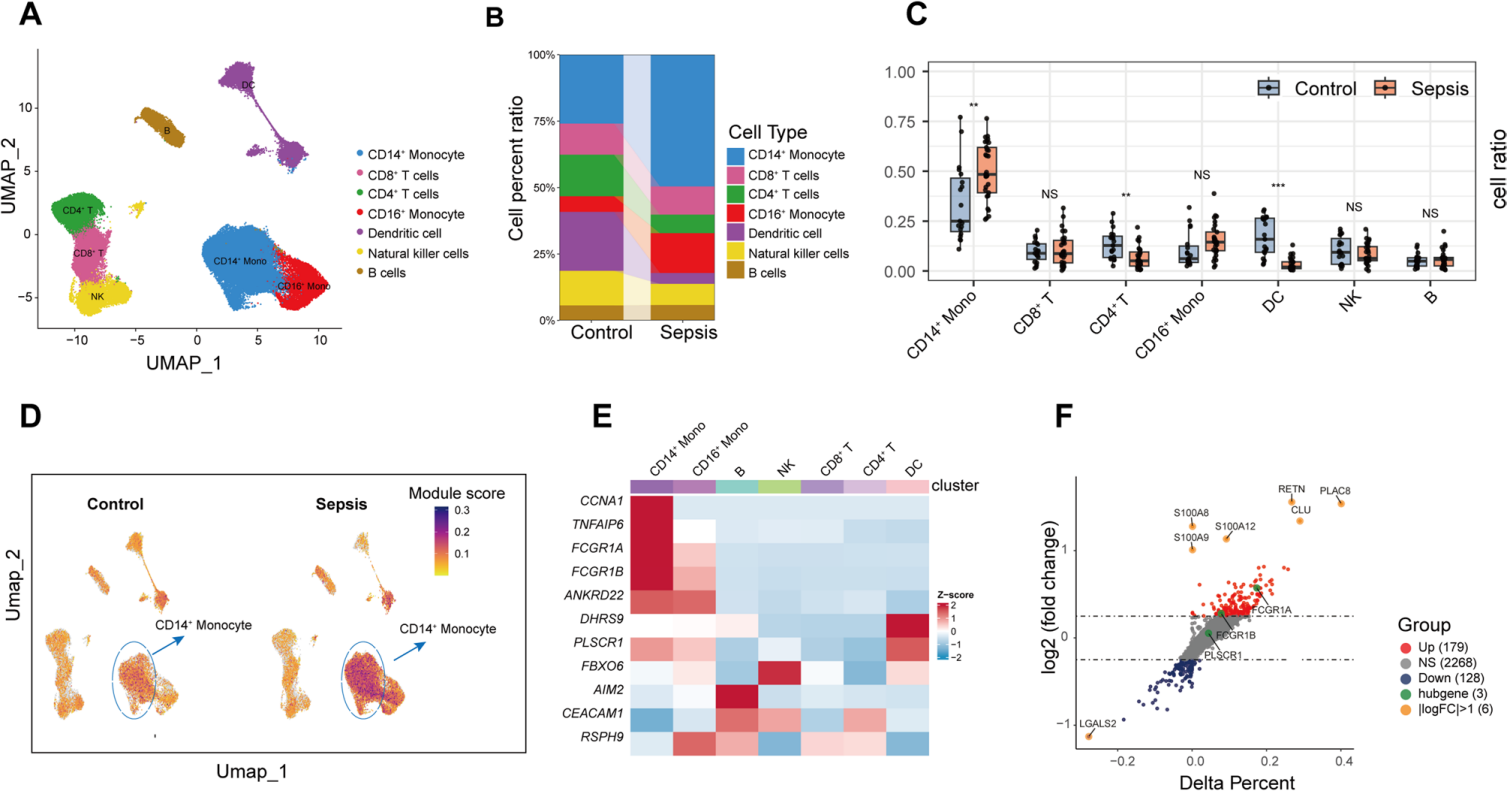
sc-RNA sequence identifies surge CD14^+^ monocytes proportion and hub gene overexpression in sepsis.** (A)** UMAP plots for each cell type, coloured by different cell types. **(B)** The average cell proportions in PBMCs from sepsis patients and healthy controls. **(C)** Comparison of cell type proportions across all samples, grouped by disease status. Each point illustrates the proportion of a particular cell type in a given sample. **(D) **Module score distribution in UMAP space for eleven hub gene modules evaluated using UCell, and grouped by disease states. **(E)** Heatmap showing the average expression of eleven hub genes between all cell types. The color scale corresponds to z-score. **(F) **Volcano plot displaying the results from differential expression analysis using the Wilcoxon rank-sum test between CD14^+^ monocytes from sepsis patients and healthy controls. The X-axis signifies the difference in individual gene detection proportions between the sepsis and control groups. Genes with log2(FC) > 1 are highlighted in orange. Statistical significance was determined using unpaired Wilcoxon rank-sum test (*P < 0.05, **P < 0.01, ***P < 0.001, NS, not significant)

To explore cellular alterations in sepsis, we performed standardized scRNA-seq analysis on a publicly available dataset (SCP548) comprising PBMCs from sepsis patients and healthy controls. Initial processing and clustering identified 16 distinct cellular populations (Supplementary Fig. 4A). Annotation using established canonical markers revealed seven major immune cell types: CD14^+^ monocytes, CD16^+^ monocytes, B cells, CD4^+^ T cells, CD8^+^ T cells, dendritic cells (DCs), and natural killer (NK) cells (Fig. 6A, Supplementary Fig. 4B). Comparative analysis of cellular composition highlighted significant differences between the sepsis and control groups. Specifically, we observed a marked increase in the proportion of CD14^+^ monocytes in sepsis patients (*P* < 0.01). Conversely, the relative frequencies of CD4^+^ T cells and DCs were significantly reduced in the sepsis cohort (Fig. 6B, C).

To assess the activity of our previously identified 11 hub genes across cell types, we calculated module scores using the UCell algorithm. UMAP projections demonstrated that CD14^+^ monocytes exhibited the highest hub gene module scores, particularly within the sepsis group compared to controls (Fig. 6D). This finding was further substantiated by heatmap analysis, which illustrated the elevated expression of these hub genes in CD14^+ ^monocytes from sepsis patients, with notable upregulation observed for genes including CCNA1, TNFAIP6, FCGR1A, and FCGR1B (Fig. 6E).

Focusing on CD14^+^ monocytes, differential gene expression analysis between sepsis patients and healthy controls identified 179 upregulated and 128 downregulated genes (applying criteria of |average log2 Fold Change| >0.25 and adjusted *P*-value < 0.05). Among the most prominent upregulated genes were *FCGR1A* and *FCGR1B*, known regulators of inflammatory responses (Fig. 6F). Consistent with these observations, an examination of the 11 individual hub genes within the CD14^+^ monocyte population across all samples confirmed generally higher expression levels in the sepsis group compared to the healthy controls (Supplementary Fig. 4C).

### Convergent and divergent transcriptional programs in CD14^+^ monocytes and CD4^+^ T cells across SLE and sepsis

**Fig. 7:**
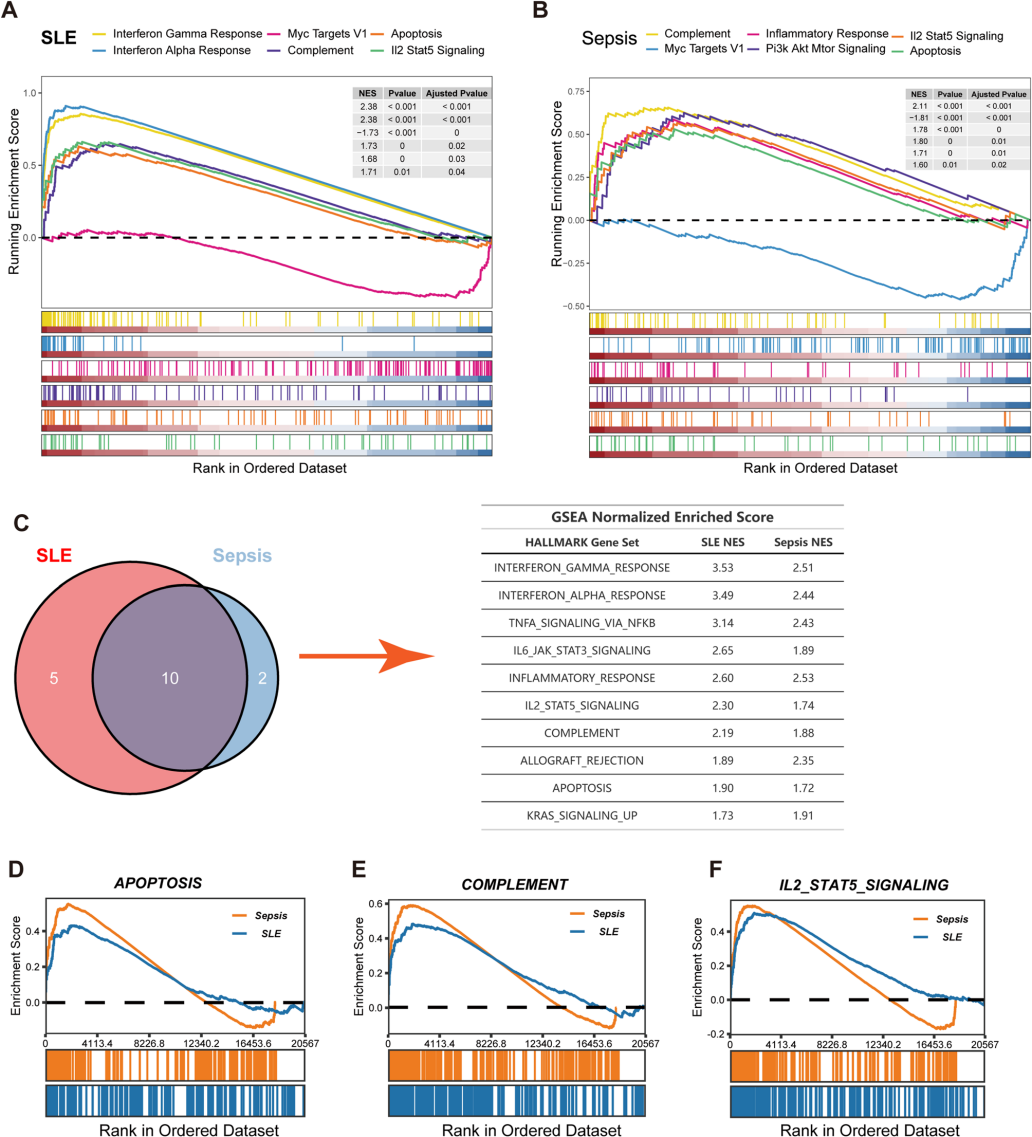
Comparison of CD14^+^ Monocyte Enrichment Analysis Outcomes in Sepsis and SLE.** (A)** Multiple-GSEA between SLE and healthy controls in CD14^+^ monocytes. **(B)** Multiple-GSEA between sepsis and healthy controls in CD14^+^ monocytes. **(C)** Venn diagram illustrating shared pathways between monocyte bulk-RNA data in SLE and sepsis cases (left), accompanied by normalized enrichment scores for these pathways (right). **(D-F)** Comparative analysis of selected GSEA pathways between SLE and sepsis

Building on the observation of expanded CD14^+^ monocyte populations and elevated hub gene expression in both SLE and sepsis, we next investigated shared and disease-specific transcriptional alterations using GSEA. In CD14^+^ monocytes from SLE patients (scRNA-seq), GSEA identified significant enrichment primarily related to interferon signaling, with the “Interferon Alpha Response” and “Interferon Gamma Response” hallmark gene sets being strongly activated (both of them NES = 2.38), while ‘Myc Targets V1’ was suppressed (NES = −1.73) (Fig. 7A, Supplementary Fig. 5A). Conversely, sepsis CD14^+^ monocytes exhibited a broader inflammatory signature, with 15 activated hallmark pathways, including prominent enrichment in “Complement”, “PI3K-AKT-MTOR Signaling”, and “Inflammatory Response” (NES = 2.11, 1.80, and 1.78, respectively (Fig. 7B, Supplementary Fig. 5B). Notably, despite these distinct dominant signatures, four hallmark pathways—“Complement”, “IL2 STAT5 Signaling”, “Apoptosis”, and “Myc Targets V1”—showed concordant regulation (activated or suppressed) in CD14^+^ monocytes from both conditions, suggesting common underlying pathomechanisms.

To validate and extend these single-cell findings, we performed GSEA on bulk RNA-seq data from purified human monocytes. Analysis of SLE monocytes (GSE148601) revealed 26 significantly activated pathways (Adjust *P* < 0.05), from which we focused on the top 15 based on enrichment significance (Supplementary Fig. 5C). In parallel, analysis of sepsis monocytes (GSE46955) identified 12 activated and 8 suppressed pathways (Supplementary Fig. 5D). Strikingly, 10 of the 12 pathways activated in sepsis monocytes were also significantly activated in SLE monocytes, strongly corroborating the overlap identified in our single-cell data (Fig. 7C). Furthermore, comparing the enrichment profiles revealed similar patterns and comparable NES for these shared pathways across both diseases and datasets (e.g., Apoptosis NES: 1.90 in SLE, 1.72 in sepsis; Complement NES: 2.18 in SLE, 1.88 in sepsis; IL2 STAT5 Signaling NES: 2.30 in SLE, 1.73 in sepsis) (Fig. 7D-F). Key inflammatory pathways like ‘Inflammatory Response,’ ‘Interferon Gamma Response,’ and ‘TNF signaling via NFκB’ also displayed parallel enrichment kinetics in both conditions (Supplementary Fig. 5F-H), underscoring shared inflammatory axes.

Expanding the analysis beyond monocytes revealed distinct systemic immune signatures. SLE patients displayed a pan-immune cell upregulation of interferon-stimulated genes (ISGs), such as IFI27, ISG15, and IFI6, consistent with a type I interferonopathy (Supplementary Fig. 6A). In contrast, sepsis patients showed broad upregulation of acute inflammation-associated genes across immune cells, particularly S100 alarmins (S100A8, S100A9, S100A12) (Supplementary Fig. 6B).

Finally, given the reduced proportion of CD4^+^ T cells observed in both diseases (Fig. 5C for SLE and Fig. 6C for Sepsis), we performed GSEA on this subset. In SLE, CD4^+^ T cells showed strong activation of “Interferon Alpha Response”, “Interferon Gamma Response”, and “Inflammatory Response” pathways (Supplementary Fig. 6C). In sepsis, while also showing enrichment for “Interferon Gamma Response”, CD4^+^ T cells were distinctly characterized by activation of “TNFα signaling via NFκB” and “Complement” pathways (Supplementary Fig. 6D), highlighting disease-specific T cell functional skewing despite similar population dynamics.

### Validation of *TNFAIP6* and *PLSCR1* expression across human conditions and murine models

**Fig. 8:**
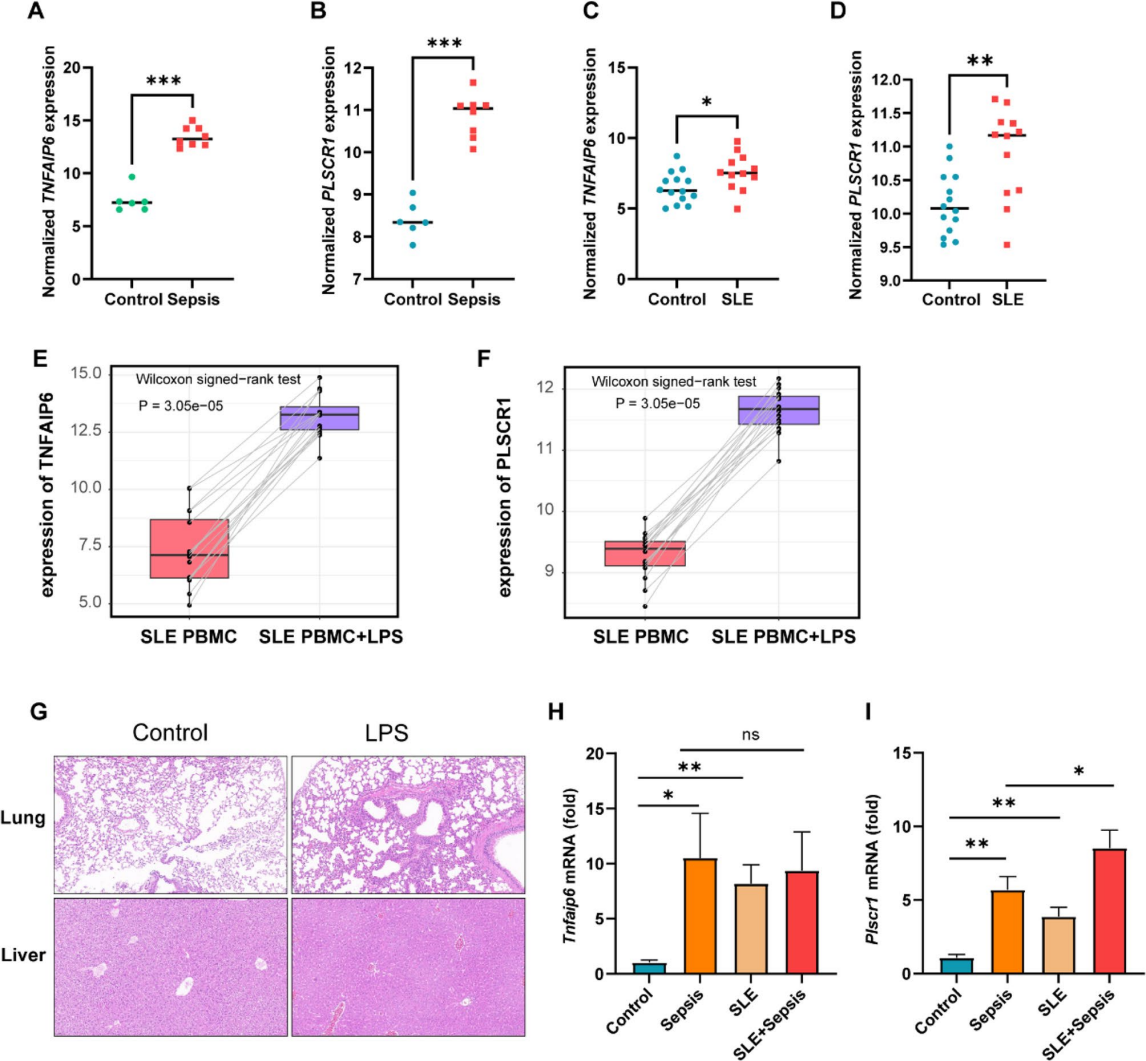
Transcriptomic Analysis and Real-Time PCR Confirm Elevated Levels of *TNFAIP6* and *PLSCR1* mRNA in Human and Mouse. (**A**-**B**) Normalized mRNA expression of *TNFAIP6* and *PLSCR1* in human monocytes from sepsis versus control, based on dataset GSE46955. (**C**-**D**) Normalized mRNA expression of *TNFAIP6* and *PLSCR1* in human monocytes from SLE versus control, derived from dataset GSE148601. (**E**-**F**) Comparison of normalized mRNA expression of *TNFAIP6* and *PLSCR1* in PBMCs from SLE patients before and after LPS stimulation with paired samples. (**G**) Representative H&E histology of lung and liver from control and LPS-treated mice. (**H**-**I**) Real-time PCR analysis of *Tnfaip6* and *Plscr1* in mouse PBMCs from control, sepsis, SLE, and SLE complicated by sepsis. Statistical significance was determined using Wilcoxon rank-sum tests across all panels: paired tests for panels E and F, and unpaired tests for panels A-D, H, and I. For panels H and I, *P*-values were adjusted using the Benjamini-Hochberg method to correct for multiple comparisons (*P < 0.05, **P < 0.01, ***P < 0.001, NS, not significant)

To validate the observed mRNA expression patterns of *TNFAIP6* and *PLSCR1*, we analyzed publicly available monocyte transcriptomic datasets. Specifically, we examined data from healthy controls alongside patients with sepsis (GSE46955) and SLE (GSE148601). Compared with monocytes from healthy controls, those from individuals with either sepsis or SLE exhibited significantly higher expression levels of both *TNFAIP6* (Fig. 8A and C) and *PLSCR1* (Fig. 8B and D). Furthermore, to assess the impact of inflammatory challenge within the SLE context, we analyzed paired PBMC samples from SLE patients available in the GSE231686 dataset. Comparing gene expression before (unstimulated) and after ex vivo lipopolysaccharide (LPS) stimulation revealed that LPS treatment significantly upregulated both *TNFAIP6* (Fig. 8E) and *PLSCR1* (Fig. 8F) expression relative to unstimulated PBMCs from the same patients.

Extending our validation to animal models, we examined PBMCs from murine models of sepsis and SLE. Following LPS injection, histopathological examination confirmed successful model establishment with significant organ injury. LPS-treated mice exhibited lung injury characterized by thickened alveolar walls, reduced air spaces, and increased cellular infiltration. Similarly, liver sections showed cellular disorganization, necrosis, and inflammatory cell infiltration (Fig. 8G). Consistent with our human data, PBMCs from both murine sepsis and SLE models demonstrated significantly elevated expression of TNFAIP6 (Fig. 8H) and PLSCR1 (Fig. 8I) compared to respective controls. Intriguingly, further analysis suggested that while *TNFAIP6* expression levels did not significantly differ between cases of sepsis with concurrent SLE versus sepsis alone, *PLSCR1* expression was significantly higher when sepsis occurred in the context of SLE. Taken together, these validation results across diverse datasets, species, and inflammatory conditions highlight the association between altered *TNFAIP6* and *PLSCR1* expression and the pathophysiology of sepsis and SLE.

## Discussion

Our integrative bioinformatic analysis identified significant molecular and cellular overlaps between sepsis and SLE. We pinpointed 11 shared hub genes (*ANKRD22*, *RSPH9*, *DHRS9*, *AIM2*, *CCNA1*, *CEACAM1*, *FBXO6*, *FCGR1A*, *FCGR1BP*, *TNFAIP6*, *PLSCR1*) potentially linking these conditions. Further analysis using LASSO regression identified *TNFAIP6* and *PLSCR1* within this set as exhibiting the highest diagnostic potential for both diseases. Notably, single-cell analysis revealed that the expression of these genes was predominantly localized to CD14^+^ monocytes, a cell population found to be significantly expanded in both SLE and sepsis compared to healthy controls. Furthermore, GSEA highlighted a striking convergence of dysregulated inflammatory and immune response pathways within these monocytes across both diseases. Our experimental validations, using human PBMCs stimulated ex vivo and murine models, corroborated several of these findings. Notably, we observed elevated *PLSCR1* levels in our experimental model combining pristane-induced autoimmunity and subsequent LPS challenge. Collectively, these findings highlight potential shared monocyte-associated molecular signatures and pathway alterations between SLE and sepsis.

Among the 11 identified hub genes, *TNFAIP6* and *PLSCR1* were selected for prioritized investigation based on their robust diagnostic potential scores observed in our initial multi-dataset screening. Their potential roles in pathogenesis further warranted this focus. TNFAIP6 (TSG-6), an acute-phase glycoprotein induced by pro-inflammatory cytokines, has complex functions, potentially reflecting both intense inflammation and attempted tissue protection [34]. Its upregulation in monocytes could signify a critical shared response element in SLE’s autoimmune milieu and sepsis’s acute inflammation. PLSCR1 influences signaling cascades and gene expression beyond its role in membrane dynamics. Its established link to SLE diagnostics and monocyte ratios [35, 36], combined with our finding of distinctly elevated expression in LPS-stimulated SLE PBMCs and SLE-sepsis mouse models (compared to sepsis alone), strongly suggests PLSCR1 dysregulation is particularly relevant at the intersection of these diseases, potentially amplifying the inflammatory response. While other shared genes like *AIM2 *[37], *ANKRD22 *[38], or *FCGR1A*/*FCGR1BP* contribute significantly, our focused validation underscores the central involvement of *TNFAIP6* and *PLSCR1* in the shared monocyte-driven pathology.

The convergence of aberrantly activated pathways within CD14^+^ monocytes further underscores their central role in linking SLE and sepsis. The observed hyperactivation of complement, interferon (alpha and gamma) responses, TNF signaling via NFκB, and IL6-JAK-STAT3 pathways in monocytes from both patient groups points to a common axis of immune dysregulation [39–41]. This shared signature implies that monocytes in SLE, potentially primed by chronic IFN exposure [42], might exhibit a heightened or altered response profile when encountering septic triggers, mediated through pathways like TNF and IL6 signaling. Such interactions could mechanistically explain the increased risk and potentially altered clinical course of sepsis in SLE patients. While transcriptomic shifts occurred in other PBMCs, the consistency and magnitude of changes within CD14^+^ monocytes strongly position them as a critical cellular nexus between these diseases.

It is noteworthy that although neutrophils are well-established contributors to the pathogenesis of both SLE and sepsis, technical limitations in our current analysis precluded a detailed investigation of this specific cell population, which could influence the observed relative monocyte proportions [43, 44]. Furthermore, these findings derive from a single dataset (GSE135779) and may reflect characteristics specific to that cohort. Given SLE’s heterogeneity, including potential variations like monocytopenia, validation in larger, diverse cohorts using multiple methodologies is crucial to confirm the broader applicability of these monocyte-centric observations.

The consistent upregulation of TNFAIP6 and PLSCR1 across diverse validation settings suggests their potential utility as biomarkers reflecting the inflammatory interplay between SLE and sepsis. However, a critical factor for clinical utility, particularly for early diagnosis of infection-induced sepsis in SLE patients, is the sensitivity and kinetics of their induction. While TNFAIP6/TSG-6 is known as an acute-phase reactant with relatively rapid induction, the precise time course of upregulation for both TNFAIP6 and, particularly, PLSCR1 in monocytes following an infectious challenge within the specific context of SLE remains undefined by our current study. Our findings demonstrate association with established disease states or post-stimulation, but future longitudinal studies are imperative to characterize how quickly these genes respond to an acute septic event in SLE patients. Determining these induction kinetics is essential to evaluate their true potential as early and sensitive biomarkers for sepsis complications in this vulnerable population.

Despite the insights gained, this study has limitations. Our analyses relied on publicly available datasets, which may introduce heterogeneity and might not fully capture the clinical complexity, especially for SLE patients developing sepsis. Consequently, direct validation in a well-characterized, prospective cohort of SLE patients, specifically monitoring for sepsis development, is crucial. Although we identified significant cellular and pathway overlaps, the precise molecular mechanisms driving the specific PLSCR1 elevation in SLE with sepsis and the exact functional consequences of the co-dysregulation require further experimental elucidation.

## Conclusion

Our study reveals shared hub genes and convergent pathways in CD14^+^ monocytes linking sepsis and SLE. *TNFAIP6* and *PLSCR1* emerged as key validated markers, with *PLSCR1* notably elevated in SLE-sepsis models. These findings implicate CD14^+^ monocyte dysfunction as a crucial immunological bridge. While potential biomarkers, *PLSCR1* requires further study of induction kinetics and mechanisms to guide diagnostics and therapies for sepsis in SLE.

## Supplementary Information

Supplementary Material 1.

Supplementary Material 2.

Supplementary Material 3.

## Data Availability

No datasets were generated or analysed during the current study.

## Abbreviations

SLE: Systemic lupus erythematosus
GEO: Gene Expression Omnibus
DEGs: Differentially expressed genes
GO: Gene ontology
WGCNA: Weighted gene co-expression network analysis
LASSO: Least absolute shrinkage and selection operator
ROC: Receiver operating characteristic
GSEA: Gene Set Enrichment Analysis
