# Supplementary material for: Bulk and single-cell transcriptome analysis reveal shared key genes and patterns of immune dysregulation in systemic lupus erythematosus and sepsis
Source: Mol Med. 2025 Dec 30;32:18. doi: 10.1186/s10020-025-01350-y (PMC12888134; doi:10.1186/s10020-025-01350-y)
Supplement: Supplementary file 1 — Supplementary Material 1. [file 10020_2025_1350_MOESM1_ESM.docx]

Supplementary Table 1. Clinical and demographic characteristics of SLE patients across datasets

| **Dataset** | **Age(years)ᵃ** | **Female, n(%)** | **Race/Ethnicity, n(%)** | **SLEDAIᵇ** |
| --- | --- | --- | --- | --- |
| GSE49454 | 38 (18-70) | 53 (85.0) | White 55 (89.0) Black 4 (6.0) Asian 3 (5.0) | 5 (0-26) |
| GSE148601 | 25.9±3.9 (21-31) | 5 (71.4) | Not provided | 6.1±6.9 (0-17) |
| GSE231686 | 50 (22-75) | 7 (87.5) | White 5 (62.5) Asian 3 (37.5) | 2 (0-8) |
| GSE135779 | 17 (10-63) | 38 (92.6) | Hispanic 18 (43.9) White 5 (12.2) Black 14 (34.1) Asian 4 (9.8) | 4 (0-19) |
| GSE228066 | 45 (14-69) | 45 (100) | Asian 45 (100) | 10 (2-33) |
| GSE61635 | Not provided | Not provided | Not provided | Not provided |
| ᵃ Age is presented as median (range) for all datasets except GSE148601, which is presented as mean±SD (range). | | | | |
| ᵇ SLEDAI (Systemic Lupus Erythematosus Disease Activity Index) scores are presented as median (range) for all datasets except GSE148601, which is presented as mean±SD (range). | | | | |
| Notes: | | | | |
| 1. Percentages are calculated based on the total number of valid data in each dataset. | | | | |
| 2. Hispanic is an ethnicity classification rather than a racial classification. | | | | |
| 3. Missing information in some datasets is marked as "Not provided". | | | | |
