# Supplementary material for: Bulk and single-cell transcriptome analysis reveal shared key genes and patterns of immune dysregulation in systemic lupus erythematosus and sepsis
Source: Mol Med. 2025 Dec 30;32:18. doi: 10.1186/s10020-025-01350-y (PMC12888134; doi:10.1186/s10020-025-01350-y)
Supplement: Supplementary file 2 — Supplementary Material 2. [file 10020_2025_1350_MOESM2_ESM.docx]

**Supplementary Table 2. Demographic and Clinical Characteristics of Sepsis Patients Across Multiple Datasets**

| **Dataset** | **Age (years)^a^** | **Gender, Male (%)** | **SOFA Score ^b^** | **Sample Collection Time ^c^** |
| --- | --- | --- | --- | --- |
| GSE95233 | 67 (59-77) | 160 (63%) | 11 (9-14) | 24-72 hours |
| GSE57065 | 62 (56-76) | 55 (67%) | Not provided | 0-48 hours |
| SCP548 | 64.5 (53.6-70.6) | 20 (69%) | 3 (1.8-4.6) | 39.7 (29.2-54.8) hours |
| GSE185263 | 61 (44-73) | 198 (57.9%) | 2 (0-4) | Not provided |
| GSE46955 | Not provided | Not provided | Not provided | Not provided |
| ^a^ Age is reported as median [Q1-Q3] in years for all datasets except SCP548. Data for SCP548 represents the weighted median [Q1-Q3] calculated using data from the primary dataset.  ^b^ SOFA score is reported as median [Q1-Q3] for all datasets except SCP548. Data for SCP548 represents the weighted median [Q1-Q3] calculated using data from the primary dataset.  ^c^ Sample collection time is presented as a range for most datasets. For SCP548, the value represents the calculated weighted average [Q1-Q3] time point based on data from the primary dataset.  Notes:  1. Percentages are calculated based on the total number of valid data in each dataset.  2. Missing information in some datasets is marked as "Not provided". | | | | |
