# Supplementary material for: Bulk and single-cell transcriptome analysis reveal shared key genes and patterns of immune dysregulation in systemic lupus erythematosus and sepsis
Source: Mol Med. 2025 Dec 30;32:18. doi: 10.1186/s10020-025-01350-y (PMC12888134; doi:10.1186/s10020-025-01350-y)
Supplement: Supplementary file 3 — Supplementary Material 3. [file 10020_2025_1350_MOESM3_ESM.docx]

**
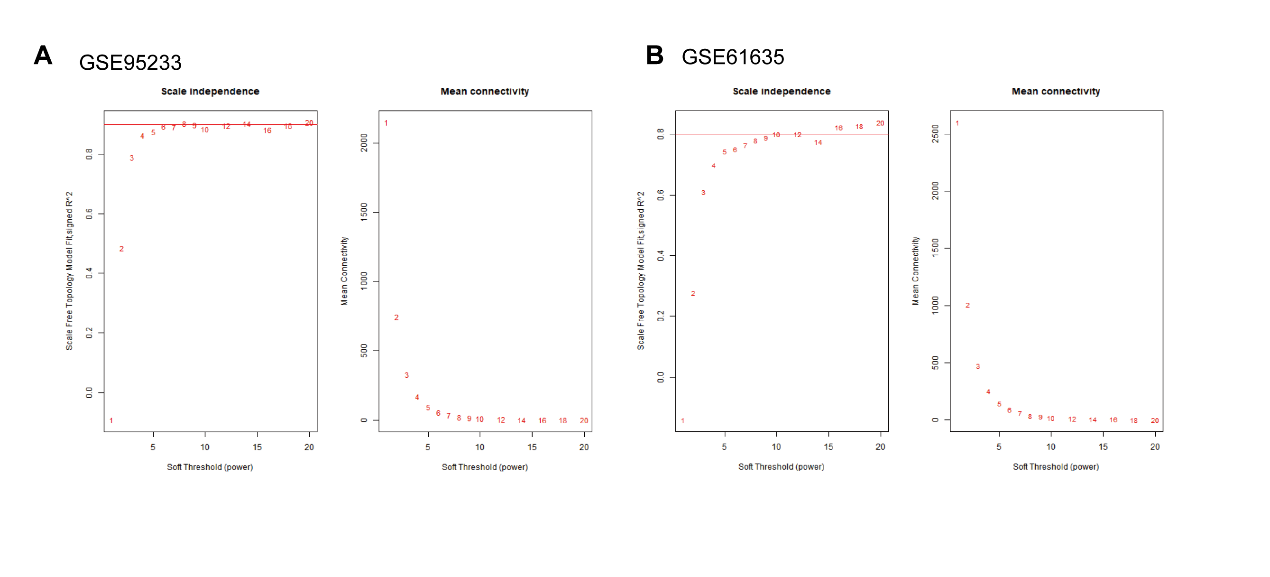
**

**Supplementary Figure 1:** **Selection of soft threshold for WGCNA.** **(A)** The scale independence and mean connectivity of GSE95233. **(B)** The scale independence and mean connectivity of GSE61635.


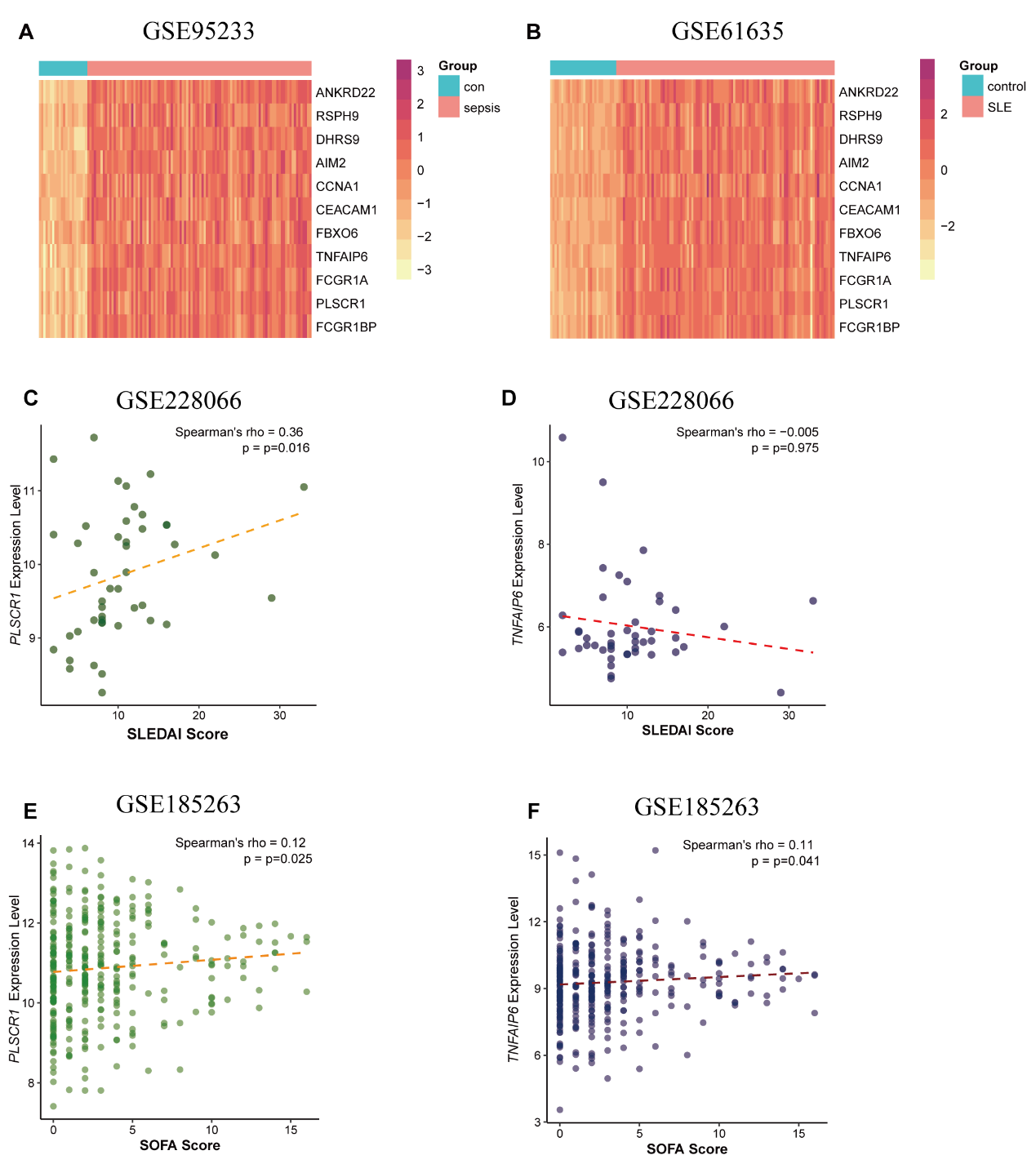


**Supplementary Figure 2: Hub gene expression analysis in sepsis and SLE datasets with correlation to disease severity.** (A) Heatmap showing hub gene expression comparing sepsis patients to healthy controls in GES95233. (B) Heatmap showing hub gene expression comparing SLE patients to healthy controls in GSE61635. (C) Scatter plot demonstrating significant positive correlation between PLSCR1 expression and SLE Disease Activity Index (SLEDAI) score in the SLE cohort (GSE228066). (D) Scatter plot showing no significant correlation between TNFAIP6 expression and SLEDAI score in the SLE cohort (GSE228066). (E) Scatter plot revealing modest but significant positive correlation between PLSCR1 expression and Sequential Organ Failure Assessment (SOFA) score in the sepsis cohort (GSE185263). (F) Scatter plot demonstrating modest significant positive correlation between TNFAIP6 expression and SOFA score in the sepsis cohort (GSE185263).


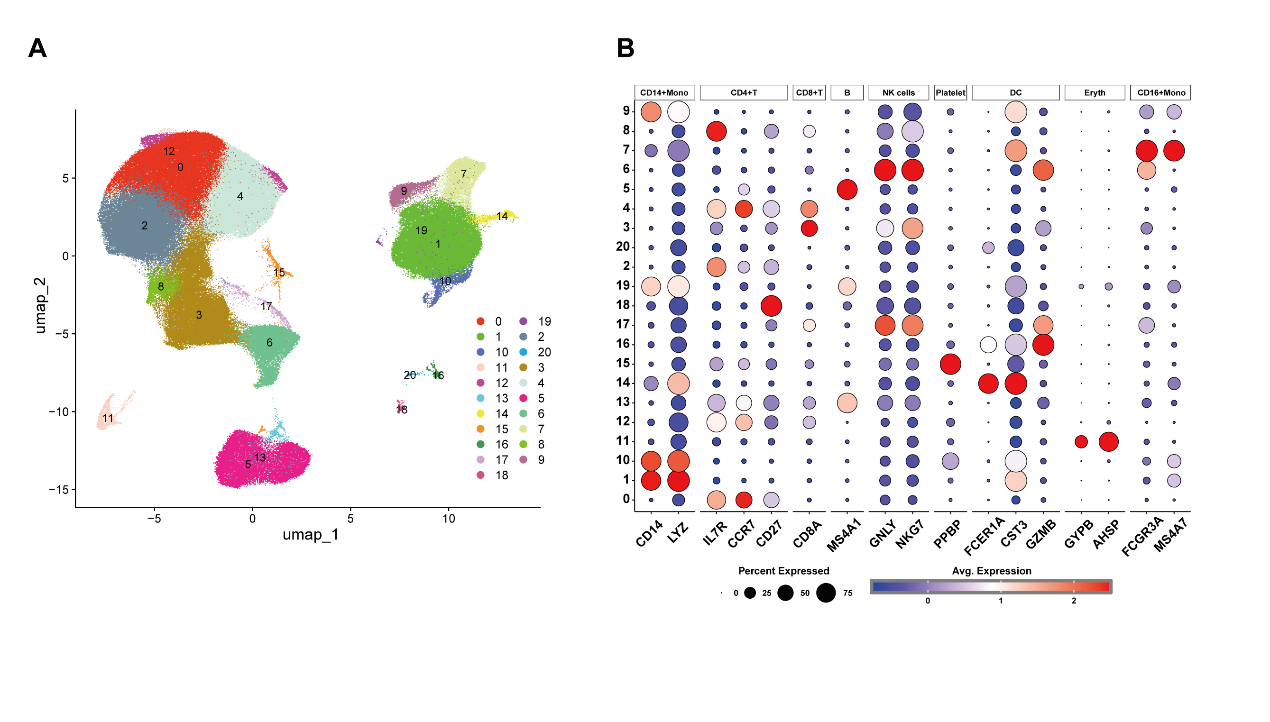


**Supplementary Figure 3: Clustering and Annotation Markers Characterization in SLE Single Cell Dataset GSE135779.** (A) UMAP plots representing cell clusters of PBMCs from dataset GSE135779, each uniquely coloured to represent distinct clusters. (B) Annotation markers for the cell clusters. The X-axis denotes specific markers for each cell type, whereas the Y-axis corresponds to individual cell clusters.


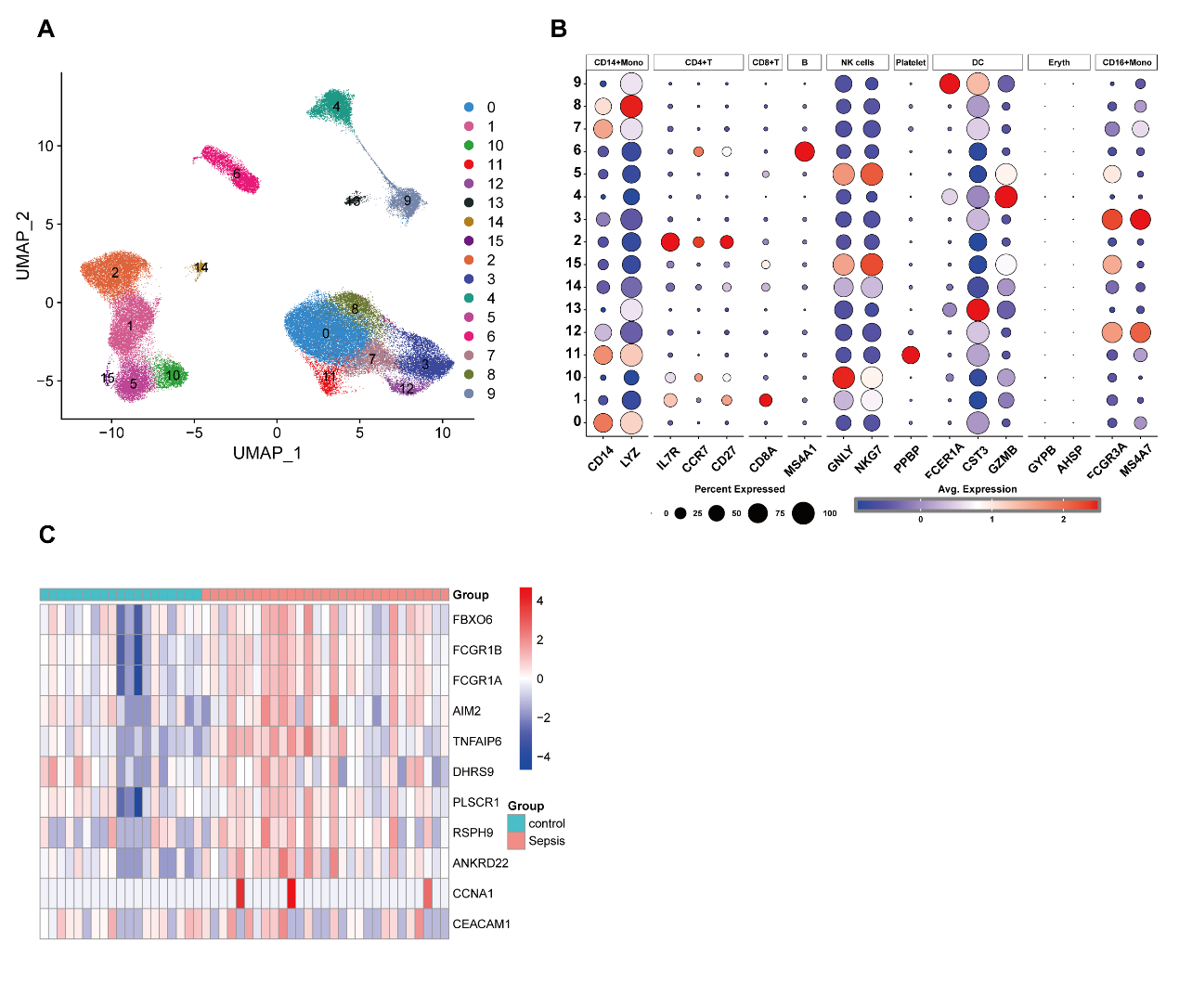


**Supplementary Figure 4: Cell Clusters, Annotation Markers, and Hub Genes Expression in the Sepsis Single Cell Dataset SCP548.** (A) UMAP plots depict PBMC cell clusters from the dataset SCP548, each colour-coded uniquely to differentiate between clusters. (B) Annotation markers associated with each cell cluster. The X-axis presents the specific markers for each cell type, and the Y-axis corresponds to the individual cell clusters. (C) Heatmap of eleven hub genes' expression across all samples, the colour scale corresponds to the z-score.


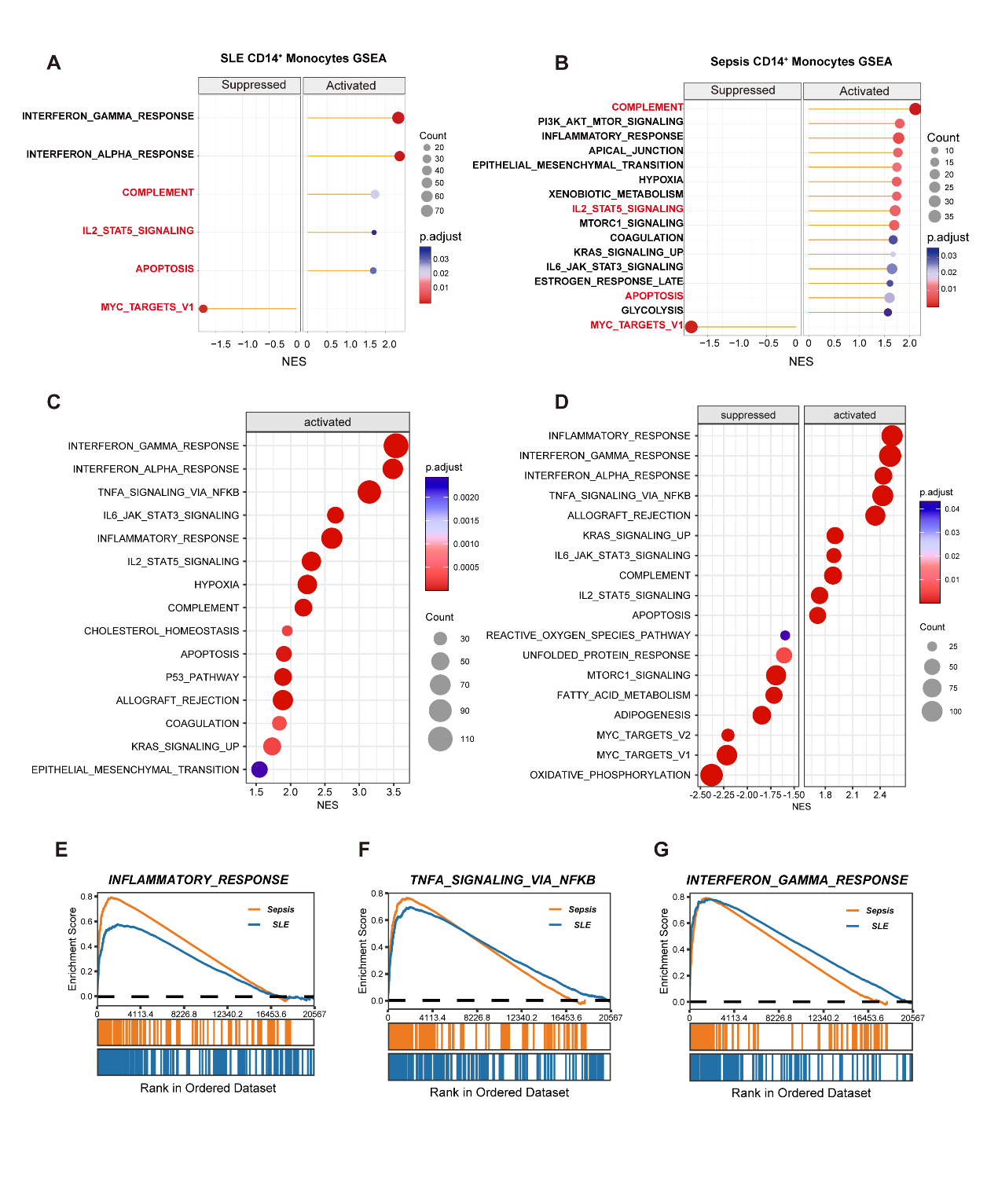


**Supplementary Figure 5: Comparative Gene Set Enrichment Analysis (GSEA) of CD14^+^ Monocytes in SLE and Sepsis Relative to Controls. (A)** GSEA results for CD14^+^ monocytes derived from single-cell transcriptomic data in SLE. **(B)** GSEA results for CD14^+^ monocytes derived from single-cell transcriptomic data in sepsis. **(C)** Top 15 enriched pathways in GSEA analysis of monocytes using bulk-RNA transcriptomic data from SLE cases. **(D)** GSEA outcomes of monocytes derived from bulk-RNA transcriptomic data in sepsis cases. **(E-G)** Comparative analysis of selected GSEA pathways between SLE and sepsis, as specified.


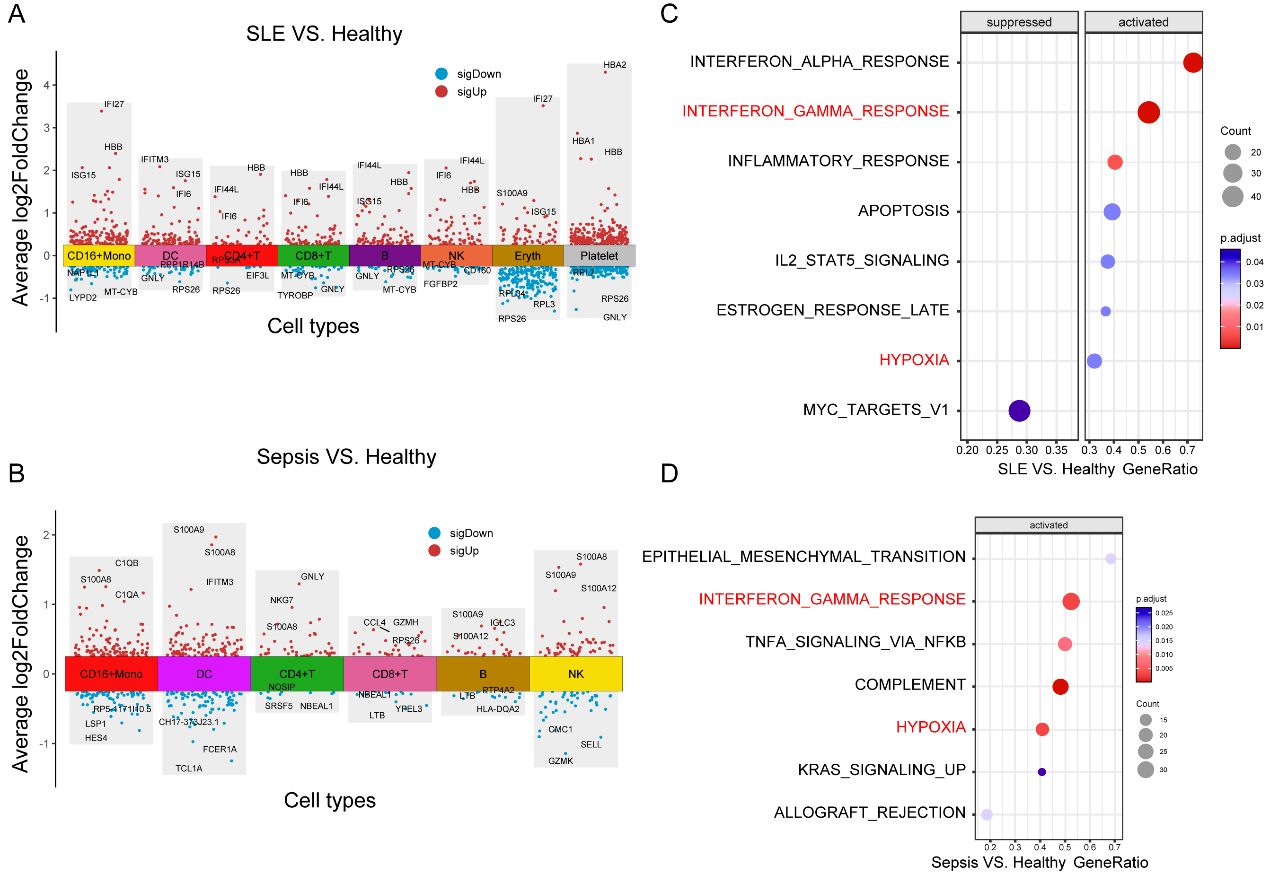


**Supplementary Figure 6:** **DEGs in various PBMC cell types among SLE, sepsis, and healthy controls, and GSEA in CD4^+^ T cells. (A)** DEGs between SLE and healthy controls across different cell types as indicated in the figure. **(B)** DEGs between sepsis and healthy controls across different cell types as indicated in the figure. **(C)** GSEA results for CD4^+^ T cells derived from single-cell transcriptomic data in SLE. **(D)** GSEA results for CD4^+^ T cells derived from single-cell transcriptomic data in sepsis.
